# Practicing security: securitisation of transboundary rivers by hydrocrats in Himalayan South Asia

**DOI:** 10.1007/s10708-023-10836-3

**Published:** 2023-02-16

**Authors:** Harsh Vasani

**Affiliations:** School of International Development, University of East Anglia, Norwich, UK

**Keywords:** Securitization, Hydropower, Brahmaputra, Dams

## Abstract

This paper examines the intersection of regional geopolitics and the governance of transboundary rivers using the case studies of multipurpose reservoirs in Himalayan South Asia. It uncovers the various ways Indian hydrocracy uses its institutional and technical expertise to strengthen India’s centrality in Nepal’s water and hydropower sectors. The practices of security undertaken by the hydrocrats are classified as structural, institutional, and statutory acts. By focusing on practices of an epistemic community like hydrocrats, this paper addresses longstanding weaknesses of the securitisation theory of being elitist and ignoring the agency of mid-level bureaucrats. It also highlights the constructivist nature of international politics. The findings contribute empirically to securitisation theory’s ‘Paris School’ of thought.

## Introduction

In 1953 Nepal received its first airfield at a site in Kathmandu called Gauchar. This airfield was built using Indian development assistance and would go on to become an international airport later renamed the Tribhuvan International Airport. The airfield made international aviation to Nepal easier. However, Indian engineers—presumably instructed by the officials in the Ministry of External Affairs—designed the runway just short enough that flights from other countries and across the mountains could not land at the airfield (read: Chinese flights). For the next seven years, India continued to administer and maintain the airport with such a tight hold that calls to the airport’s control tower were routed through the Indian embassy’s switchboard (Mihaly, 2002). In doing this, New Delhi ensured that it retained its centrality in Nepal’s geography and polity. Following the 1962 war between India and China, India forced Nepal to retract the tender of the Asian Development Bank-financed Kohalpur-Banbasa Road from a Chinese contractor.^1^ Similarly, in Nepal’s water resources sector, regional geopolitics play an influential role. For instance, in the 1960s, India’s Trishuli and Phewa hydropower projects competed with China’s Sunkoshi and Seti projects (Pun, 2008). More recently, the 1200 MW Budhi Gandaki hydropower has been rescinded twice after being awarded to a Chinese development company.^2^ Likewise, the West Seti project handed to the China Three Gorges Corporation in 2011 failed to operationalise and has been given to India’s National Hydroelectric Power Corporation as of September 2022.^3^ The geopolitical tug-of-war continues to date in the landlocked Himalayan country with the resurgence of hydropower globally, even though ambitious projects often fail to materialise. It is little wonder then that Prithvi Narayan Shah, the last ruler of the Gorkha Kingdom and the first King of Nepal, once called Nepal the yam between two boulders.

Nepal sits prominently in Himalayan South Asia. It has over 6000 rivers, 5358 lakes, and an annual run-off of over 200 billion cubic meters (Bharati et al., 2014; Dev Acharya et al., 2019). Home to the Himalayan ranges, Nepal’s glacial-fed Himalayan rivers contribute 70 per cent of the Ganges water during the dry season (December–May). According to some estimates, it has the capacity to generate 42,000 MW of hydroelectricity (Alam et al., 2017). Nepal’s geography and topography with its steep slopes and the gradient of its rivers are seen by some government officials in Nepal and India as ideal for hydropower projects. However, despite its abundant water resources, Nepal’s hydropower sector is vastly underutilised, so much so that it imports electricity from India to meet its domestic needs (Poudyal et al., 2019).

Indian policymakers see the water flowing from Nepal into India as a way to address the water crisis in India. According to estimates, 54 per cent of India faces extremely high water stress, 600 million Indians are the risk of surface water supply disruptions, and roughly 200,000 Indians die annually due to lack of access to safe water (NITI Aayog 2019; Shao et al. 2015). To be sure, the problem lies with over-extraction and the unsustainable use of water. For instance, of the total groundwater extracted by India, 89–91 per cent is used for irrigation (FAO, 2022). Water use in the agriculture sector in India is inefficient with an average Indian farmer using 2–4 times more water to produce a unit of major food crop than farmers in China or Brazil (Dhawan, 2017).

Instead of addressing the unsustainable demands and the over-extraction, Indian hydrocrats, with backing from the political class, are seeking to address the problem from the supply side. They see it as a part of their ‘hydraulic mission’ to dam the rivers, control nature, and not let a drop of water flow into the ocean without first being put to work. Turton (2003: 11) defines hydraulic mission as the “official state policy that seeks to mobilise water as a foundation of social and economic development”. Hydrocracy is the mid-level administrators, engineers, consultants, and other officials working in the various government agencies and ministries that deal with water resources and hydropower. Mirumachi (2015: 07) defines hydrocracy as the “governmental agencies responsible for the use, development and conservation of the water resources.” Molle et al. (2009) argue that the public investments in irrigation that became common in the early 20th century led to the creation of hydrocracies. Molle et al., (2009 328): define hydrocracy as “a cadre of professionals, most frequently civil engineers staffing hydraulic bureaucracies”. According to Wester (2008: 10), the hydrocracy is characterised by its “high-modernist worldview” that is set out to “control nature and ‘conquer the desert’ by ‘developing’ water resources for the sake of progress and development.” This belief is apparent in large sections of the Indian hydrocracy and is leading to the planning and construction of dams not only domestically, but also on rivers shared with neighbours. According to the national register of large dams, there are 411 dams under construction in India as of June 2019 (CWC, 2019). Numerous such projects are on transboundary rivers that run across neighbouring countries—some of whom have territorial disputes with India. Additionally, none of the South Asian states are signatories to the United Nations Watercourses Convention (UNWC), making cooperation on these international rivers difficult.

Riparian relations and governance of shared water resources are not immune to territorial disputes, and geopolitical conflicts. For instance, Warner (2012) illustrates how the regional geopolitical tensions permeated onto hydropower projects (sometimes violently) such as Ilisu and Ataturk dams in Turkey, and how different actors used water issues as leverage to gain territories in the Euphrates-Tigris basin. Warner (2012) states that when the Kurdish Worker’s Party started attacking Turkey’s hydraulic projects, Southeast Anatolia—the region where the dam project was set to be built—was placed under martial law. Warner confirmed the position of Buzan et al. (1998) that security can be contagious—securitised non-water issues may lead to water security issues and vice versa. In the South Asian context, the Indian Prime Minister alluded to using water resources flowing from India into Pakistan as a punitive measure following the 2016 Uri terror attacks.^4^ During the Doklam stand-off of 2017 between India and China, the latter stopped sharing hydrological data with India, citing renovation of their monitoring stations. Nevertheless, Beijing shared data from the same stations with Bangladesh during the same period (Khadka, 2017). The Himalayan region is a seismically active zone, and this lack of data could prove critical in times of natural disasters.

In this context, this paper examines the intersection of security with the water resources sector in the region, specifically the Himalayan South Asian region of India, Nepal, and China. I use the case study of multipurpose dams in the region to understand the several ways Indian hydrocrats use their technical and institutional knowledge to securitise the governance of transboundary rivers. Using the data gathered from fieldwork, I illustrate the distinct ways Indian hydrocrats practice security. I categorise these practices as structural, institutional, and statutory acts. The rationale for this categorization is to provide a framework to analyse the processes of securitisation in other fields through professionals of security. It follows the template set by Fischhendler (2015) for classifying securitising moves. The focus of the paper is on studying the practices of Indian hydrocrats. Hence, counter-securitisation or attempts to desecuritise the governance of the shared rivers by Nepali or Chinese hydrocrats are not discussed in the paper. The practices identified and the process of securitisation are discussed in the sections below.

The reasons for looking at securitisation through hydrocrats are multi-fold. Firstly, using such a constructivist, actor-oriented approach provides a post-structuralist lens to the study of international politics that treats state behaviour as dynamic, not static, and provides agency to actors. This follows the tradition set by securitisation scholars such as Buzan et al. (1998), Floyd (2006, 2010), Aradau et al. (2006), and Balzacq (2011) among others. Secondly, it demystifies the ‘state’ as a rational unitary actor and provides an alternative view of state behaviour that is defined and practised by epistemic communities. These epistemic communities use their technical knowledge and expertise to assist decision-makers in identifying national interests (Haas, 1992). Haas (1992) defines epistemic communities as “a network of professionals with recognized expertise and competence in a particular domain and an authoritative claim to policy-relevant knowledge within that domain or issue area”. What sets these epistemic communities apart are shared values, culture, causal beliefs, and a common policy enterprise. The tendency to look at transboundary water politics through structuralist IR theories (neo-realism, neoliberalism for instance) is criticised by certain scholars (see Furlong, 2006). Warner and Zeitoun (2008) pointed out there is scope for IR theories to engage meaningfully with questions of transboundary water politics especially if one were to look at the critical and constructivist scholarship on the topic. The scholarship of ‘hydro’ scholars and their framework of hydro-hegemony and hydro-diplomacy illustrates the range of international relations beyond the conventional theories of neo-realism and neo-liberalism (See Cascão & Zeitoun, 2010; Warner et al., 2017; Zeitoun et al., 2017; Zeitoun & Mirumachi, 2008; Zeitoun & Warner, 2006; Zeitoun & Allan, 2008). By focusing on the agency of an epistemic community, this paper tries to expand on constructivist literature on ‘hydro-politics’. Lastly, by focusing on the practices of hydrocrats, an attempt is made to move away from an elitist understanding of securitisation that overemphasis the role of elite actors and the impact of their discursive practices. Practices can be defined as “a routinized type of behaviour which consists of several elements, interconnected to one another” (Reckwitz, 2002). Floyd (2016) argues that the study of securitisation through speech acts suffers from a “constructivist deficit” where the success of securitisation is decided by scholars. A “radically constructivist” theory on security would appreciate the role of professionals of security (ibid). Similarly, Baysal (2020) argues that Copenhagen School (CS) is fixated on “macro-level discourses while ignoring micro-level practices”. Trombetta (2014), Zajko (2015), and Mirumachi (2015) try to study these micro-level practices in their research looking at climate-induced migration and security within the EU, Canada’s cyber security, and transboundary water politics in the developing world. This paper will follow their tradition and expand on the literature on security practices by epistemic communities—in this case, the hydrocrats.

## Methodology

This paper is the result of fieldwork conducted in New Delhi between September 2020 and February 2021. Officials from various Indian ministries and other government offices were interviewed (see Appendix 1). The China factor in India-Nepal relations is well-known and so is the regional geopolitics. Hence, I ventured into the field to understand the causes behind the lack of progress on large, multipurpose projects in the region. However, what I observed were the several ways, Indian officials, using their agency, and technical and administrative expertise tried to address what they perceive as threats to India’s centrality in the region’s energy politics or threats to India’s water resources. In the process, these actions managed to securitise various aspects of transboundary water governance in the region. Interviews with the Indian hydrocrats informed this paper. These interviews were semi-structured and were conducted in person and virtually. The objective was to map the perspectives of the institutions (see Appendix 1) on factors that affect transboundary water resource governance between India and Nepal. Transcripts of these interviews were then uploaded onto NVIVO software and then coded for thematic analysis. An attempt was made to go beyond the semantics of the data and find latent themes in it and the data was interpreted to find patterns, meanings, and implications of the security processes (see Braun et al. 2019; Braun and Clarke 2006). Interviewees were identified using minutes of the meetings available on the Nepali Government websites of Pancheshwar and SKSK projects, LinkedIn, and snowballing method.

I draw on the framework built by Baysal (2020) and Fischhendler (2015) to illustrate the practices of security exercised by Indian hydrocrats. This framework regards securitisation as a process and emphasises security practices as equally important as speech acts. Going by the traditional theoretical framework of Buzan, Wæver, and Wilde (1998), it is also unclear who is the ‘audience’ that needs to be persuaded by the securitising actors. Balzacq (2011) did point out that given the thickness of security programs, the lines between the securitising actor and the audience tend to blur. A look at the skewed gender ratio in hydrocracies reveals the masculine nature of these organisations. Of all the participants in this study, only three were women. This included one Additional Secretary in the Indian Ministry of Agriculture, one journalist covering the energy sector in India, and one private sector consultant. The near-total absence of women in hydrocracy surely must impact policy prescriptions and governance.^5^ An assessment of the impact of this masculine hydrocracy could be an interesting avenue for future research.

## Securitisation theory: practices and professionals

Securitisation is defined as the move “that takes politics beyond the established rules of the game and frames the issue either as a special kind of politics or as above politics” (Buzan et al., 1998). It is also called an extreme version of politicisation (Buzan, Wæver & Wilde 1998). By securitising an issue, the securitising actor allows the suspension of the usual protocols and procedures and adopts extraordinary measures. Successful securitisation has three components: existential threats, emergency action, and breaking free of established rules (ibid). However, some passages from Buzan et al., (1998) suggest that for successful securitisation, extraordinary (or emergency) measures may not necessarily be adopted; and only an argument for an existential threat may be made (by the securitising actors). These existential threats should be persuasive enough to build a platform for emergency measures. As we will be in the later sections, in the case of the dam-for-dam approach on the Brahmaputra, it is difficult to ascertain any ‘extraordinary measures’. The act of building a dam as a response to an upper riparian’s project is extraordinary in itself. Securitisation is a social constructivist theory, meaning the threat is subjective. Unlike classical realism in international relations which examines the balance of power between states, neorealism, as advanced by Kenneth Waltz (1979) and John Mearsheimer (2001), focuses on security instead of power and how the pursuit of security impacts state behaviour. However, unlike neorealists, securitisation falls neatly under the post-structuralist camp. In other words, the emphasis is not on the overarching structure under which states exist, but on the actors who have some agency and the ability to influence state behaviour.

The various units in securitisation—especially if done using discourse or speech acts—involve the referent object, the securitising actor, and functional actors. The referent object is seen to be existentially threatened and has a legitimate claim to survival, the securitising actor securitises the issue by declaring something (the referent object) as existentially threatened, and the functional actor is one who affects the “dynamics of the sector” or the one which “significantly influences the decisions in the field of security” (Buzan et al., 1998). As we will see in the later sections, on the river Brahmaputra, the construction of a Chinese dam near the ‘great bend’ is seen as an *existential threat* to the *referent object*—the river’s biodiversity and water security for local communities living downstream. The *securitising actors* here are the hydrocrats who have planned a dam downstream of the Chinese dam to offset the latter’s impact on the water flow as well as give the Indian side prior rights to a continued flow of water under the UN Watercourses Convention. *Functional actors*, in this case, are the various NGOs, advocacy groups, and local communities that affect the proposed dam in certain ways. It must be noted that not all cases of securitisation may have functional actors. This is particularly true if the region does not have a strong or vocal civil society or if the securitising move has been unanimously accepted by the audience.

The governance of transboundary rivers is studied using the framework of securitisation theory by various scholars. For instance, Mirumachi (2013) has argued that the Indian government used its “technical and institutional expertise” to frame a securitised discourse on the Mahakali river that forms the western border between Nepal and India to construct the Tanakpur Barrage. During the negotiations over the Tanakpur project, the Indian government managed to securitise it by claiming that the survival of both states was at stake (ibid). Securitisation, in this case, was the result of speech acts from the Indian Prime Minister when he wrote to his Nepali counterpart asserting the necessity of the Tanakpur project to protect Indian territory from inundation and erosion during monsoons (Bhasin, 2005). Beyond the study of discourse, Mirumachi (2015) studied the role of official hydrocracy in securitising transboundary water governance. She asserts the importance of studying the hydrocracy since it “accumulates vast amounts of knowledge and information through their use of technical expertise about potential river development projects that inform the state agenda” (Mirumachi, 2015). Similarly, Ho et al. (2019) used the Q methodology to understand the perceptions of ‘water experts’ in India and China on the riparian relations between the two states. However, their assertion that India and China have tried to desecuritise their shared water resources owing to China’s desire to “stabilise its southern periphery, expand bilateral trade and investment opportunities with India, and reduce India’s alignment with the United States” and India’s reluctance in antagonising China owing to the power asymmetry is problematic (ibid: 265). A closer look at the activities of China and India in the region—be it in Nepal (discussed below) or on the Brahmaputra—demonstrates the complicated and securitised riparian relations. The problem perhaps lies with the discourse analysis using Q methodology and the view of securitisation that is over-reliant on discursive actions rather than micro-level security practices. Furthermore, research on the securitisation of water resources between India and China limits themselves to the case of the Brahmaputra River (for instance: Rampini, 2021; Sahu & Mohan, 2021). While recent developments on the Yarlung Tsangpo-Brahmaputra make it necessary for the study of securitisation, there are other avenues in the region that illustrate the securitisation of water resources (discussed in the following sections).

The reliance on speech acts as the primary securitising move in the traditional, CS-dominant, view of the theory is seen by certain scholars as elitist (Baysal, 2020; Bigo, 2008; Bigo & McCluskey, 2018; Booth, 2007). In other words, such an approach places disproportionate importance on high-level decision-makers whilst ignoring the ‘professionals of security.’ The scholars who place significance on the professional of security are together categorized as the ‘Paris School’ on security (Diskaya, 2013; Floyd, 2006; Wæver, 2004). These scholars focus on the impact of security knowledge and technology on the political structure (Aradau et al., 2006) The professionals of security are the officials whose actions lead to the construction of security issues (Baysal, 2020). Baysal (2020: 13) defines them as individuals who “obey the rules and orders and implement the decisions taken at a higher level, acting within the security definitions of the high-level decision-makers.” In the case of military security, these could include “soldiers, intelligence agents, or militants” (ibid: 13). While speech acts assist in understanding how some issues are presented as a security threat, securitization is a social and political construct that depends on other forms of actions as well. Securitisation could be a result of everyday practices of government officials who may not enjoy the same forms of “capital and legitimacy” as high-level decision-makers. (Bigo & Tsoukala, 2008: 4–5). According to some scholars, securitisation has a lot to do with “mundane bureaucratic decisions of everyday politics, with Weberian routines of rationalisation, of management of numbers instead of persons, of use of technologies” (Bigo & Tsoukala, 2008: 5). Some of the securitisation moves practised by the bureaucracies or the media are so routinised and institutionalised that they escape scrutiny or are never discussed (Bigo & Tsoukala, 2008). This paper focuses on the professionals of security—the hydrocrats—who work under the structure set by the decision-makers, to implement and further the agenda of security using their technical and institutional knowledge and expertise.

## Structural acts

**Fig. 1:**
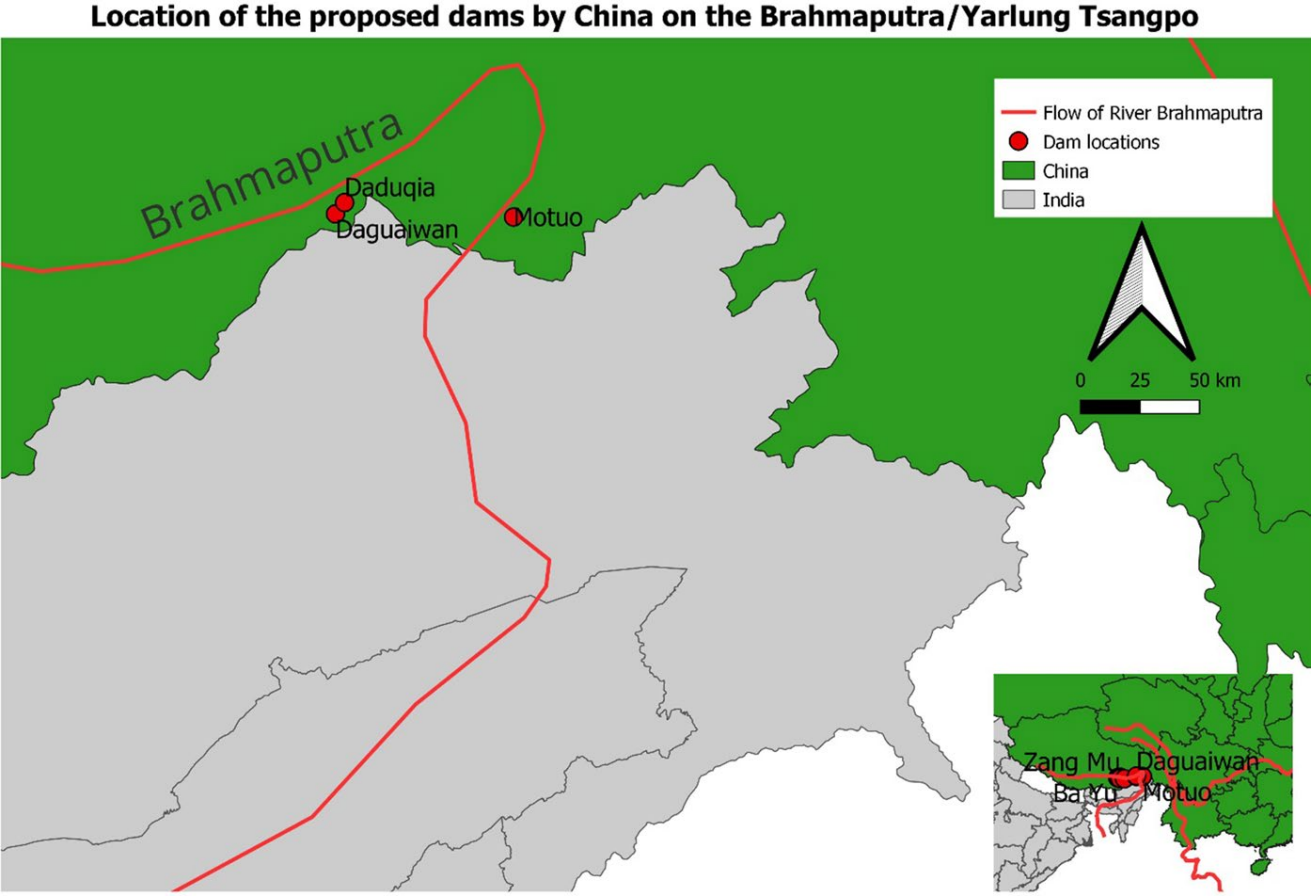
Location of dams on the Brahmaputra River, *Source: created by the author*

**Fig. 2:**
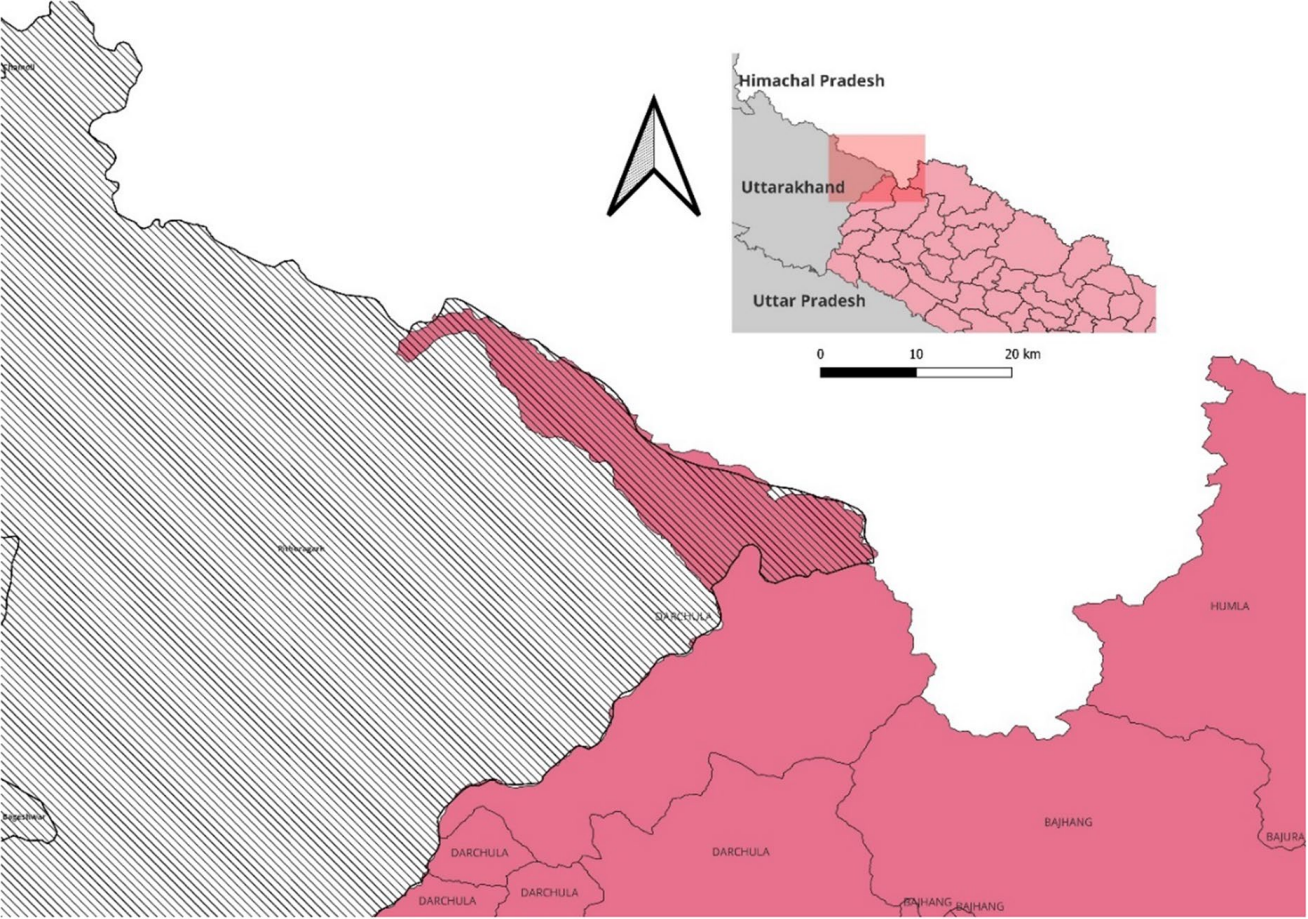
The disputed territory of Kalapani, Lipulekh, and Limpiyadhura that is claimed by India and Nepal (territory stripped in pink), *Source: created by the author* .

**Fig. 3:**
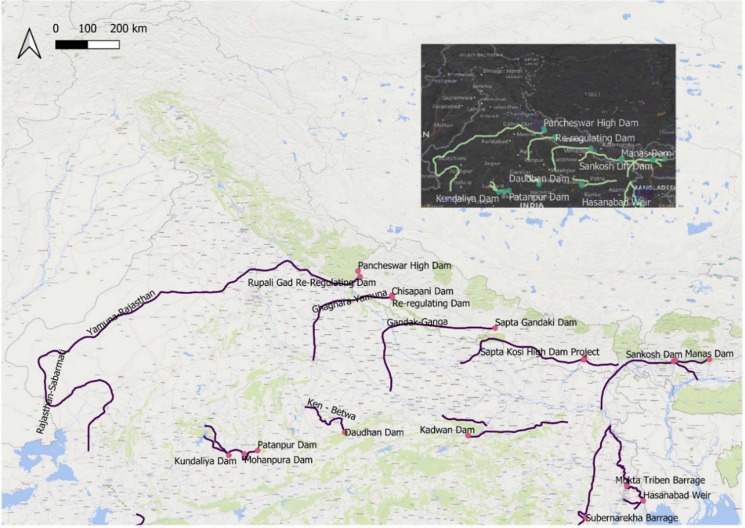
Himalayan component of the interlinking of Indian rivers with storage reservoirs in Nepal and India, *Source: created by the author using geospatial data from Higgins et al. *(2018)

Structural acts are actions with physical and material results that are meant to allude to or address security threats. For instance, building roads or airstrips at international borders. These allow quick and easy mobilization of armed forces to the border, make supplies to these forces possible, and display the seriousness of the state to defend its sovereignty. The results of these actions—border roads, airstrips, naval bases, and the construction of bunkers—are physical and tangible. In the context of this research, the structural act is the plan to build a dam by a section of Indian hydrocrats in Arunachal Pradesh on the Siang River to mitigate the impact of the upstream dam by China. Indian hydrocrats believe that China’s dam on the river (called Yarlung Zangbo in China) could potentially impact the flow of water in downstream Arunachal Pradesh and Assam.^6^ The declaration of the dam by officials in India’s Central Water Commission illustrates their view of Chinese construction close to the border through a securitised prism. The dam of concern—close to the river bend that enters India—is reported to be a 60 GW hydropower project on the lower section of the Brahmaputra River that is three times the size of the Three Gorges Dam. Almost immediately an official from India’s Jal Shakti Ministry announced India’s plan to build a multipurpose project in Arunachal Pradesh that will offset the impact of the Chinese dam. This project is called the Upper Siang project and is envisioned as a 9.75 GW hydropower project on the Siang, the principal constituent river of the Brahmaputra (CWC, 2021). (Figs. 1, 2 and 3).

In a report tabled in the Indian Parliament, Indian hydrocrats stated that they believe dams close to the ‘great bend’, even if they are run-of-river projects, can impact the flow of water in Brahmaputra and possible water diversion by China can cause water insecurity along the river (Ministry Jal Shakti, 2021). With a dam, Indian hydrocrats believe, China can regulate the flow of water and can cause floods. The report provides insights into the threats Indian officials perceive from China’s ambitions to dam the Yarlung Tsangpo/Brahmaputra. While Indian political leaders have so far refrained from speaking about the dam as a security threat, there are structural arrangements (or *acts*) designed by hydrocrats that allude to the ‘threats’ posed by the Chinese dam on the great bend to India’s environmental security. The timing of the project and the statements issued by officials in India’s Central Water Commission indicate that one of the main purposes of the project is to counter the impacts of the Chinese dam.^7^

Other than countering the physical impact of the Chinese dam upstream, officials in various Indian government ministries side has also been looking to strengthen its prior use rights over the Brahmaputra (Bhaskar & Ghosh, 2010). Under Articles 5 and 6 of the UN Watercourses Convention (UNWC), a state building a dam upstream should utilise the resource in an “equitable and reasonable” manner that does not impinge upon the “existing and potential uses of the watercourse” by another [downstream] state (UN, 1997: 4–5). Building a dam downstream gives the Indian side legal cover for the continued flow of water, and the right to utilize the watercourse, and mandates China to cooperate and consider the interests of the downstream states (India and Bangladesh). It is worth noting while India is building the dam to strengthen its prior use rights under the UNWC, it is not a signatory to the convention (and neither is China). Additionally, even if India seeks to have the legal cover of UNWC, it assumes that China would adhere to the principles laid out by international laws. The functional actors in this scenario are the NGOs, advocacy groups and the local communities that affect the status of the dam. While various advocacy groups have criticised the project on environmental grounds, local communities view the Chinese dam upstream as a threat (Parashar, 2017; PTI, 2021). It is difficult to ascertain what, if any, are the extraordinary measures in this securitising move. It is understood that the pre-feasibility study of the project is under way *after* the project has been approved. However, in the knotty and often chaotic hydropower sector in India, it is not unusual for feasibility studies to be re-conducted if the previous assessments have expired.^8^ This cements the assertion made by Buzan et al. (1998) that extraordinary measures may not be necessary even if a securitising move has been made. To take another example, while conscription or levying of taxes are ‘extraordinary measures’, the securitising actor(s) may decide not to take these measures in case the state already has sufficient soldiers in the armed forces or if the state treasury has enough resources. The negation of these measures does not invalidate securitising moves.

## Institutional acts

Institutional acts of securitisation are actions made to or within an institution as a response to an external threat or seeing the institution as the referent object that is being threatened. The exclusion of certain institutions from public scrutiny or transparency reflects the securitised nature of that institution. For instance, removing an institution from the remit of acts like the Freedom of Information Act (the UK) or the Right to Information Act (India) or making budgetary allocations to certain institutions (intelligence agencies) classified or confidential. By taking these institutions out of public scrutiny, the securitised nature of either the institution or certain actions undertaken within those institutions is indicated. Other instances of institutional acts of securitisation could be the exclusion of civil society from decision-making processes or the inclusion of armed forces, diplomatic corps, or intelligence agencies in institutions *outside* of their usual remit (Fischhendler, 2015). The institution in question could well be the referent object that is under threat. For instance, the outbreak of the SARS-CoV-2 (COVID-19 virus) was seen by states across the world not only as a (biological) threat to public health but also as a threat to their public health institutions like the National Health Service (UK) itself. The securitisation of the pandemic—where the referent objects were public health, and the associated institutions like the NHS—allowed for extraordinary measures like public lockdowns, mandatory health screening, quarantine, including military and paramilitary forces in anti-COVID operations etc.

In the context of this research, the Pancheshwar Multipurpose Project, to be developed jointly by Nepal and India, has gained “strategic” importance since it is viewed by Indian hydrocrats as crucial to food security in the Ganga plain and an important link in the ambitious interlinking of Indian rivers project.^9^ The project is the centrepiece of the 1996 Mahakali treaty between India and Nepal. Under the treaty, both sides sought to build a dam upstream of the Tanakpur Barrage on the Mahakali River that forms Nepal’s western border with India. Indian hydrocrats see this project as essential not only for food and water security but lately also for national security. The Pancheshwar project seeks to ensure year-round irrigation of land under the Sharada command (1.61 million hectares) by providing water in the dry season (WAPCOS, 2017). It is also a link in the Himalayan component of the river interlinking plan. The river interlinking plan has been on the drawing board since the 1970s with various governments seeking to act upon the ambitious plans. Prime Minister Narendra Modi, however, has gone ahead with the Ken-Betwa River linking project at a cost of Rs. 460 billion with five other such river-linking projects being finalised (Sharma, 2022).

The Kalapani trijunction, where the Mahakali River is believed to originate is claimed by both India and Nepal.^10^ The trijunction is seen by Indian officials as strategically important since it borders China, and sovereignty over it can help in the speedy mobilisation of troops to the border (PTI, 2020). Additionally, for Indian policymakers, the Kalapani region offers an advantageous position as it is located at an altitude of approximately 20,000 feet and can be used as an observation post overlooking Chinese territory (Subramanian, 2020). Sovereignty over the Kalapani trijunction depends on the origins of the Mahakali River, and the origins of the river itself are disputed by the governments of India and Nepal. Nepal’s western boundary with India was marked out of the Treaty of Sagauli between the East India Company and Nepal in 1816, following the Anglo-Nepalese War of 1814–16. According to the treaty, territory west of the river lies with Nepal while territory east of the river belonged to then British India (Gyawali & Dixit, 1999). The differing interpretation of the source of the river has caused territorial disputes. Indian side believes that the river originates in the Kalapani region at an elevation of about 7820 m and is part of Uttarakhand’s Pithoragarh district. In contrast, the Nepali side states that the river originates either in Limpiyadhura (15 km from Kalapani) or in Lipulekh and is part of its Dharchula district (Jha, 2020; Rising Nepal, 2020; Shukla, 2019). Some account suggests that the river originates from a stream in Limpiyadhura, northwest of Lipulekh—thus making Kalapani, Limpiyadhura and Lipulekh, fall east of the river and part of Nepali territory (Subramanian, 2020). Whereas the Indian position is that the source of the river is well below the Lipulekh pass, and while the Sagauli treaty does not demarcate the area north of the springs, administrative and revenue records from the nineteenth century show Kalapani on the Indian side and considered as part of present-day Pithoragarh district in Uttarakhand (ibid).

The institutional act of securitisation here is the inclusion of the diplomatic corps and armed forces in agencies and institutions meant to deal with the Pancheshwar project and the escalation of the Pancheshwar Development Authority (PDA) to the diplomatic level. The PDA, an “independent, autonomous” bilateral body established to “finalise the detailed project report” and expedite implementation of the project, has in its governing body officials of foreign affairs ministries along with Ambassadors of both sides.^11^ The breakdown of negotiations over the Pancheshwar project has also led to intervention by India’s Ambassador to Nepal. Such an intervention by the Ambassador was categorized as important due to the “strategic” nature of the project.^12^ There has been a shift in the justification of the project as well. While official documents, as well as personal communication with hydrocrats from India and Nepal, reveal that the project is primarily aimed at storing water for temporal and spatial transfer intended for irrigation in the Sharada command in Northern India, a statement by a minister in the Govt of India from 2020 suggests flood control is being touted as the primary aim of the project (ANI, 2020). This shift in project justification seems deliberate since any flood moderation benefits from Pancheshwar were stated to be “incidental” at best (WAPCOS, 2017). Furthermore, the armed forces too intervened in the project either directly or by expressing concerns over the river’s origin and the sovereignty over Kalapani. The project was on the agenda of the Chief of the Indian Army when he visited Nepal in 2020 (Bhalla, 2020; IANS, 2020). The Army Chief also suggested that Nepali officials have been protesting the Indian road to Lipulekh pass “at the behest of someone else”.^13^

The interventions by top-tier Army officials, the inclusion of diplomatic corp in the PDA, and interventions by the Ambassador can be classified as *extraordinary measures.* The referent objects, in this case, are food and water security in the Ganga plain, and territorial sovereignty over the Kalapani trijunction. The threats are the loss of augmented water that will be used to provide year-round irrigation of land under the Sharada command (1.61 million hectares) by providing water in the dry season, as well as the loss of the Pancheshwar component of the river interlinking project. The institution (PDA) is seen as a tool to respond to threats and the securitising act is its escalation to diplomatic levels and interventions by armed forces. (Table 1)

## Statutory acts

Statutory acts of securitisation are legislative or legal provisions that practice security. The acting agency or institution seeks to signify particular security threats from a source to a referent object using its powers to declare statutes or laws. In 2016, India’s Central Electricity Authority (CEA) issued guidelines that termed electricity trade as a matter of “strategic, national and economic importance” (CEA, 2016: 03). The guidelines also laid down conditions that made cross-border electricity trade difficult for Nepal (and Bhutan). The guidelines allowed “participating entities” to trade electricity from only those generation projects owned or funded by the Government of India, Indian public sector undertakings (PSUs) or private companies with 51% or more Indian ownership. The guidelines allow Indian companies to import electricity from generators owned or controlled by the government of the neighbouring country after seeking a one-time approval. These two clauses give preferential treatment to Indian entities and made investments in Nepal’s hydropower sector by any third party (read: China) uncertain and financially precarious. Large hydropower projects in Nepal depend on Indian acquiescence to buy surplus electricity since energy consumption in Nepal is low. In effect, these guidelines make India the sole possible foreign investor should Nepal agree to develop large hydropower projects. The governments of Nepal and Bhutan vehemently protested these guidelines and as a result, the updated guidelines removed the restrictive clauses (Ministry of Power, 2018). However, in February 2021, the CEA issued “procedures” for any entity to import or trade electricity from a generating station located outside of India using the Indian electricity grid. These procedures have repeated the restrictive clauses that make regional electricity trade dependent on New Delhi’s consent and deny the use of Indian grids by any electricity generation projects owned or controlled by any third country sharing a “land border” with India (CEA, 2021: 15).

The trade of electricity creates regional interdependencies that India can use to assert its political and economic centrality in the region since inter-regional trade would not be possible without using Indian infrastructure. Indian hydrocrats have been protective of their geoeconomic position in South Asia—especially in Nepal. These guidelines reaffirm India’s centrality—something New Delhi views as being *threatened—*by discouraging trilateral trading of electricity or regional groupings among South Asian states that could stand up to India.^14^ According to one former Joint Secretary in India’s Ministry of Power, Chinese investments in Nepal’s hydropower could replace India as the sole economic actor in the sector.^15^ These guidelines, hence, offset Chinese influence in the energy and water resources sectors of Nepal.^16^ The *extraordinary measure* that makes the issuing of these restrictive guidelines a *securitising move* is that they go against the spirit of energy cooperation as endeavoured by Indian officials and politicians. India has proclaimed ambitions of global grid connectivity to offset the need for energy storage, balance electricity grids, and encourage energy transition (Modi, 2021). India also pledged at the 2014 SAARC (South Asian Association for Regional Cooperation) summit to encourage regional electricity trade. The *functional actors*, in this case, are the members of Nepali and Bhutanese civil society and government officials that protested the promulgation of the guidelines.

## Conclusion

The paper has illustrated the various ways Indian hydrocrats practice security. These practices are either to allude to what the hydrocrats perceive as a security threat or to address it. Their institutional knowledge and technical expertise are employed under the structure set by elites. How this structure is established by the elites could be a subject of another paper, but what is important to note here is the agency of the epistemic community (hydrocrats) in matters of securitisation. For instance, Indian hydrocrats used their knowledge of procedural and legal requirements of regional electricity trade to ensure India retains its centrality in the region and also remains the only hydropower developer in Nepal (statutory act). Similarly, the building of a reservoir to offset—what these hydrocrats perceive to be a threat to water security in Northeast India as well as India’s claim to prior use rights under international water law—can be seen as a result of their institutional expertise (structural act). Finally, the inclusion of diplomatic corp and armed forces in the Pancheshwar Development Authority (PDA) designed to govern the Pancheshwar project, the intervention by India’s Ambassador to Nepal in the PDA, and the Indian Army Chief’s involvement in the Kalapani issue are institutional acts of securitisation where the chief institution is the PDA.

This focus on an epistemic community helps address the criticism of the securitisation theory among a section of scholars that view the traditional view of securitisation as elitist and ignoring the Weberian routines of rationalisation. Scholars of international relations may find the role of hydrocrats in securitising and responding to securitisation interesting as it highlights the constructivist nature of international politics. Securitisation is a post-structuralist theory and this paper demonstrates it by highlighting the agency of certain actors (in this case, the Indian hydrocracy). The findings of the paper aim to contribute to the growing debate on securitisation as understood by scholars Balzacq, Bigo, and Floyd.

It should be noted that the three acts of security identified in this paper—structural, institutional, and statutory—are not mutually exclusive. A structural act (of promulgating a dam to claim prior rights) or statutory act (of publishing guidelines reinforcing the centrality of India in the region’s energy politics) are conducted within an institution (Central Water Commission and Central Electricity Authority, for instance). These two acts have a legitimate claim to be institutional in nature along with being ‘structural’ or ‘statutory’.

The practices discussed in this paper are by no means exhaustive. There may be other practices in the hydrocracy’s ‘toolbox’ to deal with matters of security that may be unexplored in this paper. Further research could reveal other methods of securitisation by hydrocrats. Moreover, this paper did not focus on countersecuritisation conducted by Nepali and Chinese hydrocrats. These could be interesting opportunities for further research.

**Table 1:** Practices of security in transboundary water resources in Himalayan South Asia

	Security Professionals	Referent object	Threat	Security practice	Functional actor	Extraordinary measures
Structural Act	Engineers at central water commission, govt of india	Food and water security in North-eastern India; legal rights over Brahmaputra water flow	Water diversion, holding back river silt by China	Building of the Upper Siang project downstream of the Chinese dam	Local communities, NGOs, and advocacy groups	N/A
Institutional Act	Diplomatic corp, armed forces	Food security in the Ganga plain, India’s river interlinking plan, and sovereignty over Kalapani	Cessation of the river interlinking and agricultural transformation plans; Losing sovereignty over the Kalapani region	Inclusion of Ministry of external affairs officials in Pancheshwar Development authority; intervention by Indian Ambassador in the project negotiations; intervention by India’s Chief of Army Staff	N/A	Escalating project planning and negotiations at the diplomatic level; Involvement of Indian Army Chief
Statutory Act	Officials at central electricity authority	Centrality of India in South Asia’s energy politics; the position of India as the exclusive economic actor in Nepal’s water resources	Trilateral or regional groupings that could negate India’s influence; Chinese investments replacing Indian investments in Nepal	placing conditions on using Indian infrastructure and regional trade	Nepali and Bhutanese government officials. The civil society of Nepal and Bhutan	Acting against the South Asian Association for Regional Cooperation pledge for regional electricity trade and against Indian ambitions for global grid connectivity

## Appendix 1

**Table:**

Organisation name	Location	Number of participants	Retired	Serving	
Central electricity authority	New Delhi	One		One	
Central water commission	New Delhi	Six	Two	Four	
National water development agency	New Delhi	One		One	
NHPC (erstwhile national hydroelectric Power Corporation)	New Delhi	One	One		
Ministry of Jal Shakti	New Delhi	One	One		
Ministry of power	New Delhi	One		One	
Ministry of new and renewable energy	New Delhi	One		One	
Ministry of external affairs	New Delhi	Four	Two	Two	
Ministry of agriculture	New Delhi	One		One	
Ganga flood control commission	New Delhi	One		One	
Water and Energy Commission Secretariat	Kathmandu	Two	One	One	
Ministry of energy, water resources, and irrigation	Kathmandu	Three	Three		
Nepal electricity authority	Kathmandu	One	One		
SJVN Arun-III Power Development company (SAPDC)	Kathmandu	One		One	
Water Resources research and development center	Kathmandu	Two	One	One	
National planning commission	Kathmandu	One	One		
Department of electricity development	Kathmandu	One		One	
Jalsrot vikas sanstha (JVS)	Kathmandu	One		One	
Pancheshwar development authority	New Delhi and Kathmandu	Four	Two	Two	

## Footnotes

1 Personal communication with former Managing Director, Nepal Electricity Authority, August 2020.

2 For details, see Prasain, S. (2022, September 2). Budhi Gandaki hydropower project registered as public company. The Kathmandu Post. https://kathmandupost.com/money/2022/09/02/budhi-gandaki-hydropower-project-registered-as-public-company.

3 See MyRepublica. (2022, June 7). Indian company to build West Seti Hydropower Project. MyRepublica. https://myrepublica.nagariknetwork.com/news/indian-company-to-build-west-seti-hydropower-project/.

4 For details, see The Indian Express. (2016, September 27). Blood and water cannot flow together: PM Modi at Indus Water Treaty meeting. The Indian Express. https://indianexpress.com/article/india/india-news-india/indus-water-treaty-blood-and-water-cant-flow-together-pm-modi-pakistan-uri-attack/

5 See for instance, Sehring et al., (2022).

6 For details, see this report tabled in the Indian Parliament: Ministry Jal Shakti. (2021). Flood Management in the Country including International Water Treaties in the field of Water Resource Management with particular reference to Treaty/Agreement entered into with China, Pakistan and Bhutan. In Lok Sabha Secretariat. http://164.100.47.193/lsscommittee/Water Resources/17_Water_Resources_12.pdf and Lok Sabha. (2018, December 26). Unstarred question no 2520 Dams on Brahmaputra by China. Government of India. http://164.100.47.194/Loksabha/Questions/QResult15.aspx?qref=75761&lsno=16

7 For instance, see Arora, N., & Ghoshal, D. (2020, December 1). India plans dam on Brahmaputra to offset Chinese construction upstream. Reuters. https://www.reuters.com/article/us-india-china-hydropower-idUKKBN28B4NN.

8 The Environmental Impact Assessments (EIA) undertaken for projects in India are valid for five years. Often times, the EIA has to be retaken due to delays in project operationalisation.

9 Personal communication, former Ambassador of India to Nepal, 09/10/2020.

10 Sovereignty over the Kalapani trijunction depends on the origins of the Mahakali River, and the origins of the river itself are disputed by governments of India and Nepal. Nepal's western boundary with India was marked out of the Treaty of Sagauli between the East India Company and Nepal in 1816, following the Anglo-Nepalese War of 1814–16. According to the treaty, territory west of the river lies with Nepal while territory east of the river belonged to then British India (Gyawali and Dixit 1999). The differing interpretation of the source of the river has caused territorial disputes. Indian side believes that the river originates in the Kalapani region at an elevation of about 7,820 m and is part of Uttarakhand's Pithoragarh district. In contrast, the Nepali side states that the river originates either in Limpiyadhura (15 km from Kalapani) or in Lipulekh and is part of its Dharchula district (Jha 2020; Rising Nepal 2020; Shukla 2019). Some account suggests that the river originates from a stream in Limpiyadhura, northwest of Lipulekh—thus making Kalapani, Limpiyadhura and Lipulekh, fall east of the river and part of Nepali territory (Subramanian 2020). Whereas Indian position is that the source of the river is well below the Lipulekh pass, and while the Sagauli treaty does not demarcate the area north of the springs, administrative and revenue records from the nineteenth century show Kalapani on the Indian side and considered as part of present-day Pithoragarh district in Uttarakhand (ibid).

11 See MoEWRI. (n.d.). Pancheshwar Multipurpose Project. Govt of Nepal. Retrieved October 7, 2022, from https://www.pmp.gov.np/acts-and-regulations.php.

12 Personal communication, former Ambassador of India to Nepal, 09/10/2020.

13 See The Indian Express. (2020, May 16). Road row: As Army Chief sees hand of third party, Nepal threatens action. The Indian Express. https://indianexpress.com/article/india/army-chief-general-mm-naravane-china-nepal-border-issue/

14 Personal communication, former Ambassador of India to Nepal, 09/10/2020.

15 Personal communication, former Joint Secretary, Ministry of Power, Govt of India, 14/10/2022 and former Ambassador of India to Nepal, 09/10/2020.

16 Personal communication, former Joint Secretary, Ministry of Power, Govt of India, 14/10/2022.

## Declarations

### Data availability

Data sharing does not apply to this article as no datasets were generated or analysed during the current study.
